# A Smart Toy Intervention to Promote Emotion Regulation in Middle Childhood: Feasibility Study

**DOI:** 10.2196/14029

**Published:** 2019-08-05

**Authors:** Nikki Theofanopoulou, Katherine Isbister, Julian Edbrooke-Childs, Petr Slovák

**Affiliations:** 1 Evidence Based Practice Unit, University College London and Anna Freud National Centre for Children and Families London United Kingdom; 2 Department of Computational Media University of California, Santa Cruz Santa Cruz, CA United States; 3 Department of Informatics King's College London London United Kingdom

**Keywords:** mental health, children, families, stress, psychological, emotional adjustment

## Abstract

**Background:**

A common challenge with existing psycho-social prevention interventions for children is the lack of effective, engaging, and scalable delivery mechanisms, especially beyond in-person therapeutic or school-based contexts. Although digital technology has the potential to address these issues, existing research on technology-enabled interventions for families remains limited. This paper focuses on emotion regulation (ER) as an example of a core protective factor that is commonly targeted by prevention interventions.

**Objective:**

The aim of this pilot study was to provide an initial validation of the logic model and feasibility of in situ deployment for a new technology-enabled intervention, designed to support children’s in-the-moment ER efforts. The novelty of the proposed approach relies on delivering the intervention through an interactive object (a *smart toy*) sent home with the child, without any prior training necessary for either the child or their carer. This study examined (1) engagement and acceptability of the toy in the homes during 1-week deployments, and (2) qualitative indicators of ER effects, as reported by parents and children.

In total, 10 families (altogether 11 children aged 6-10 years) were recruited from 3 predominantly underprivileged communities in the United Kingdom, as low SES populations have been shown to be particularly at risk for less developed ER competencies. Children were given the prototype, a discovery book, and a simple digital camera to keep at home for 7 to 8 days. Data were gathered through a number of channels: (1) semistructured interviews with parents and children prior to and right after the deployment, (2) photos children took during the deployment, and (3) touch interactions automatically logged by the prototype throughout the deployment.

**Results:**

Across all families, parents and children reported that the *smart toy* was incorporated into the children’s ER practices and engaged with naturally in moments the children wanted to relax or calm down. Data suggested that the children interacted with the toy throughout the deployment, found the experience enjoyable, and all requested to keep the toy longer. Children’s emotional connection to the toy appears to have driven this strong engagement. Parents reported satisfaction with and acceptability of the toy.

**Conclusions:**

This is the first known study on the use of technology-enabled intervention delivery to support ER in situ. The strong engagement, incorporation into children’s ER practices, and qualitative indications of effects are promising. Further efficacy research is needed to extend these indicative data by examining the psychological efficacy of the proposed intervention. More broadly, our findings argue for the potential of a technology-enabled shift in how future prevention interventions are designed and delivered: empowering children and parents through *child-led, situated interventions*, where participants learn through actionable support directly within family life, as opposed to didactic in-person workshops and a subsequent skills application.

## Introduction

### Background

Mental health conditions are the main contributor to the substantial increase in childhood disability in the last decade [1], with most having their onset in childhood or adolescence [2,3]. Recent estimates suggest a 10% prevalence of mental disorders in children and adolescents in Great Britain [4] and 12% in Europe [5], whereas approximately 1 in every 4 to 5 youth in the United States meets criteria for a mental disorder with severe impairment across their lifetime [6,7]. This realization is fueling calls for interventions in childhood to avert the development of long-term disability [8-10]. Research in prevention science showcases the feasibility of such interventions in child populations: prevention programs develop key cognitive and emotional protective factors—such as emotion regulation (ER) or coping strategies—which, in turn, can reduce the incidence of mental health disorders in later life [11-17]. There are a variety of types of prevention programs, from *universal interventions* that are designed to be used with all children to *indicated* interventions that are targeting those already presenting with early signs of serious disorders [12]. Similar to therapeutic settings, existing prevention programs rely predominantly on in-person training. As a result, these interventions struggle with the challenges of cost, reach, and intervention fidelity [18-22].

Although existing programs are relatively successful in targeting children within the *captive audience* context of schools [21-25], a principal challenge remains in extending this support into the day-to-day contexts in which protective competencies are applied, practiced, and developed [22]. The current model relies on parents to deliver such at-home interventions and requires extensive training to do so effectively: For example, a shortened version of the Incredible Years program [26,27] still required 12 to 24 weeks of parent training in groups of 6 to 10 parents for 2.5 hours, once a week. Other programs, such as the seminal Perry Preschool program, were even more intensive, comprising a 2-year program of 2.5 hours of interactive academic instruction daily for children at school, coupled with 1.5-hour weekly home visits by trained staff [28]. Such approaches experience low enrollment rates, and the lack of continued engagement with interventions beyond formal delivery classroom context is also a common limitation [18-20]. These difficulties in bridging the formal school and informal home contexts are crucial in prevention science: family interactions are a strong mediating factor for developing resilience and impacting core socioemotional competencies, especially for younger children [29-33]. Moreover, lack of consistency of at-home and at-school support diminishes the effects of prevention programs [22,34].

New delivery mechanisms and intervention approaches are sorely needed to address these issues [8]. Digital mental health interventions are increasingly seen as having the potential to deliver on these aims, revolutionizing when, how, where, and to whom interventions can be delivered [10,35-37]. Although the interest in technology-enabled mental health continues to soar—especially in the context of treatment for adult populations—a consistent set of challenges has, however, emerged around ensuring uptake and long-term engagement of digital interventions [10,38]. Reliance on didactic and information delivery models, limited use of user-centered design, and lack of immediately perceived benefits leading to low motivation are commonly cited reasons [39-41]. These difficulties are likely to be exacerbated for prevention interventions for children, but surprisingly little research has investigated it empirically [42,43]. As such, it is not clear if and how technology could be used to facilitate transfer of such learning from school into families; or to enable new types of interventions that would empower parents and children to further develop protective competencies independently of formal training programs.

### This Research

This work investigates a proof-of-concept prototype of a newly proposed intervention delivery mechanism within the context of (1) universal prevention programs [25] for children aged 6 to 10 years and their families and (2) ER as a specific instance of a psychological protective factor. We chose ER as it is a fundamental life skill, with effects on life outcomes comparable in size to those of IQ or family social status [44,45]. Research shows that these effects are wide-reaching: if ER is poorly developed, it leads to increased incidence of both internalizing and externalizing mental health disorders [46-49] and is associated with societal problems such as criminal behavior [50], low personal well-being [44], and academic underachievement [51]. Moreover, existing intervention research shows that ER is difficult to develop without detailed in situ guidance and support [26,52-54]; and parenting strategies play a key role in shaping child emotional coping and regulatory skills [55-63]. This is particularly important within underprivileged families: prior research repeatedly shows that children from these populations are at risk of low self-regulation competencies at an early age [64,65], and the gap further widens over the school years [66].

The data reported here build on an iterative user-centered design process, which led to the development of a novel intervention prototype described in the next section. Within the 2-year-long development phase (reported in full elsewhere [67]), we worked with children, parents, and prevention science experts to codesign a proof-of-concept technology platform to support children in developing ER skills. Theoretically, the intervention is grounded both in basic models of ER [68], as well as close collaboration with developers of evidence-based interventions (*Second Step*), while also deeply involving children and families in codesign to ensure the intervention fits into their daily lives [39,40,69]. In effect, the designed prototype attempts to fuse the understanding of evidence-based methods from prevention science (what works), human-computer interaction (what is technically feasible and acceptable to users), as well as insights into the everyday practices of families within the social context we designed for (what people actually do). This iterative design process has led to a novel *situated* intervention model: the intervention is delivered through an interactive object (*smart toy*) sent home with the child, without any prior training necessary for either the child or their carer (see the next section for design details and logic model).

### Feasibility Study Aims

The aim of this qualitative study was to provide an initial validation of the feasibility of core fundamental principles underpinning the proposed novel intervention model [70], which was developed in previous research [67]. Specifically, the intervention model assumes that (1) children will be naturally compelled to keep interacting with the intervention without external guidance; (2) it will become incorporated into their everyday emotion regulatory practices, even without any formal training; and finally (3) the intervention would be perceived as acceptable to parents. Given the novel nature of the proposed delivery mechanisms, it is crucial to test whether these principles are fulfilled by the current prototype before more expansive investigations take place.

Data from exploratory deployments reported in our previous study [67] are promising; however, these are limited by short post hoc interviews with children, no information from parents, no objective log data, and only very short deployment times (median 3 days). This study builds on these preliminary findings using a range of data-collection methods (pre- and postinterviews with parents and children, log data analysis, and child photo diaries) to investigate (1) engagement and acceptability of the device in the homes during 1-week deployments and (2) subjective indicators of effects on emotion regulatory practices (whether positive or negative), as reported by parents and children.

### Intervention Design and Logic

**Figure 1:**
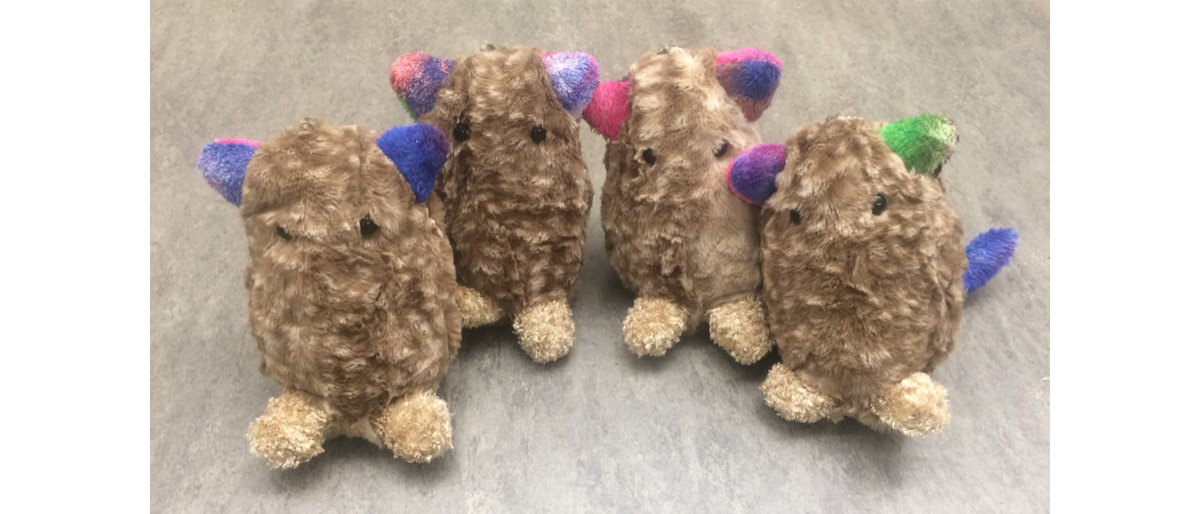
The physical prototype.

**Figure 2:**
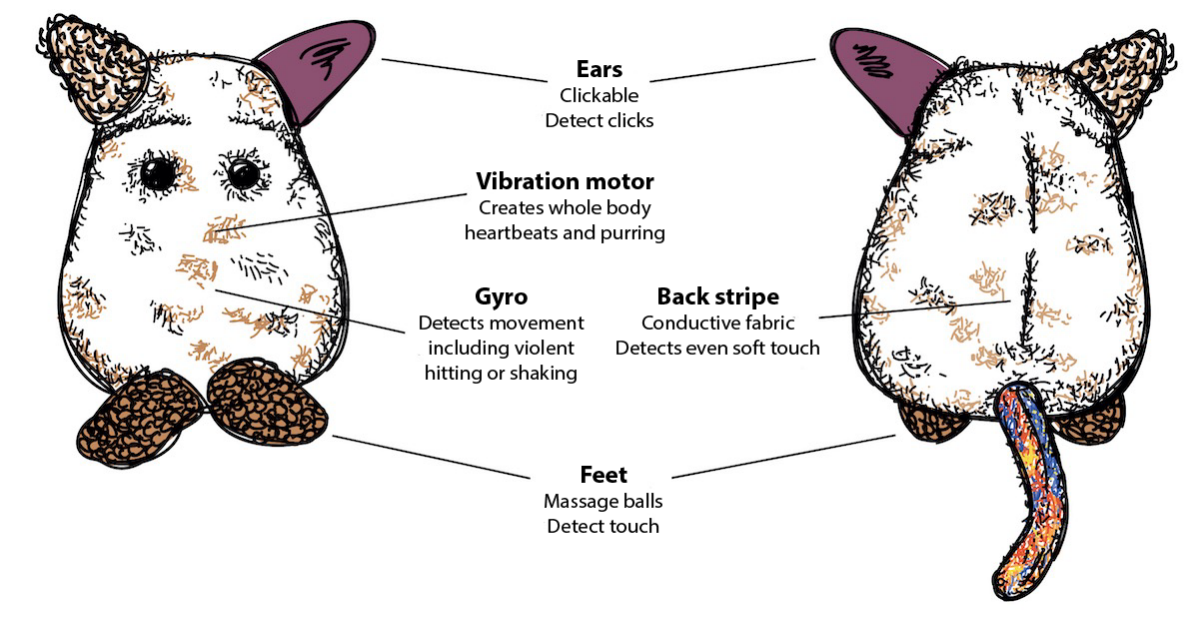
Overview of the prototype's interactive components.

The prototype takes the form of a hand-crafted plush toy (see Figure 1 and the study by Slovak et al [67] for the design process), which was designed to travel home with the child from school and support in-the-moment soothing. The toy is introduced to the child as an *anxious creature that needs kind attention from humans*, such as soft stroking and hugging. Embedded electronics enable the prototype to produce vibration patterns that simulate a heartbeat (ranging from frantic to slow and steady). When picked up, the toy emits a frantic heartbeat that slows down if the child uses calm stroking movements, as registered by the embedded sensors (see Figure 2). If the toy is *soothed* for long enough, the prototype transitions into a purring vibration indicating a calm, contented state. For a full description of the physical design, interactive features, and a more detailed logic model, see Multimedia Appendix 1. We included 29 publications in Multimedia Appendix 1 [65,68,71-99].

The logic model underlying the intervention is assumed to operate on 3 levels building on each other: Level 1 pertains to directly *providing in-the-moment soothing support* to children in naturally occurring emotional moments when they would attempt to calm down. The prototype’s physical and interaction design was aimed to tap into a number of known regulatory factors, grounded theoretically in Gross’ extended process model of ER [68]. Specifically, we designed the prototype interaction with the aim to impact 2 separate stages: the *attentional deployment stage* [71-75], by shifting children’s attention from the emotion-eliciting situation toward interacting with the toy and the *response modulation stage*, by facilitating downregulation through pleasant tactile interaction analogously to the mechanisms assumed to underpin emotion regulatory effects of human-animal interaction [76-81].

Level 2 is concerned with mechanisms that *facilitate children’s long-term engagement* with the intervention, building on the positive subjective experience of in-the-moment soothing. The framing of the toy as an *anxious creature in need of assistance* is the hypothesized key driver: we assume that this framing will not only support conveying the benefits resulting from extrinsic ER [77,82,83], but also facilitate the creation of a sense of relationship and responsibility for the *well-being* of the creature, similar to the long-term engagement seen with child-oriented robots [75] or products such as Tamagotchi [84-86].

Finally, level 3 is assumed to emerge from repeated experience of soothing interactions over time, leading to a *shift in children’s ER practices and implicit beliefs about emotion*. Specifically, we hypothesize that repeated interactions with the toy will result in the establishment of more adaptive ER patterns and shift children’s implicit beliefs about the controllability of emotion [87,88], a well-known target for intervention [89-93]. As these effects are expected to arise only through ongoing long-term interactions and, thus, rely strongly on appropriation in situ, we did not expect to see any indicative data for these proposed mechanisms within this pilot study; however, these will be crucial for long-term effect of the smart toy intervention. This study aimed to provide pilot indicative data pertaining to levels 1 and 2.

## Methods

### Overview

The goals of this early feasibility study were to investigate the engagement and acceptability of the device in the homes during 1-week deployments with children aged 6 to 10 years and their families and also subjective indicators of effects on emotion regulatory practices (whether positive or negative), as reported by the parents and children. Together, the aim was to collect indicative qualitative data pertaining to level 1 and 2 of the underlying logic model: we were interested to see if children would find the individual interactions comforting, whether they would sustain engagement over the week periods (and what role any emerging *relationship* with the toy might play here), and whether the toy will become embedded into their everyday activities, including being explicitly used for ER.

### Study Design

As we were interested in studying natural appropriation in situ, children were given a prototype, a *discovery book* that presented the simple narrative and suggested playful activities, and a simple digital camera to keep at home for 7 to 8 days. We gathered data through a number of channels. The main sources were (1) semistructured interviews with parents and children before and right after the deployment; (2) any photos the children took during the deployment, which also served as *ticket to talk* about their experiences during the week; and (3) automatically collected logs by the prototype, which recorded all touch interactions throughout the weekly deployment.

The discovery book contained some information about the *creature’s* background and various activities the child could fill in on their own or with the help of their parents, such as *photo challenges* around the toy, and an *emoji diary* where they could use emoji stickers to keep track of how they and their *creature* were feeling on each day of the deployment. In designing the discovery book and activities, our aim was to facilitate children’s engagement with the toy in a playful manner as well as complement the interview data with a richer understanding of how families experienced having the toy at home. As such, the discovery book was as much a research tool as a part of the intervention (implicitly providing the narrative and suggested activities).

The study was funded by a personal fellowship and University College London (UCL) and received ethical approval from UCL’s ethics committee (3923/005).

### Recruitment

The prototypes and accompanying materials were deployed in waves to 10 families of 11 children (3 girls, 8 boys; aged 6-10 years) from August to November 2018. One additional family had been recruited, but experienced a malfunctioning prototype and has not been included in the dataset. Participants were recruited from 3 communities in the United Kingdom through a range of methods, including online advertisements, in-person recruiting in 2 schools which had served as recruitment sites for previous phases of the project, and snowball sampling. The majority of participating families (7/10, 70%) lived in an area falling within the 20% most deprived in England (measured according to English Indices of Multiple Deprivation [100]), with the remaining 30% (3/10) living in areas falling within deciles 3 to 5. Recruitment was stopped based on data saturation [101]: the interview data collected were highly consistent across families, with only limited new insights emerging by the tenth interview, within the context of a pilot study.

### Procedure

All engagements with families were conducted by the first author, who holds an MSc in developmental psychology. The researcher visited families who had orally agreed to take part to obtain consent from parents and assent from children, conducted a semistructured interview with at least 1 parent, and gave children the toy, discovery book, and a simple digital camera to keep at home for 7 to 8 days (1 deployment was extended for a day because of a technical failure and 2 more for scheduling reasons). The first semistructured interview with parents focused on families’ existing emotion regulatory practices, perceived challenges to ER, and parents’ expectations from the week-long deployment. After 3 or 4 days, the researcher visited families again to change the toy’s battery. On the last day of the deployment, the researcher visited the families to pick up the toy and materials and interview each child and at least 1 of their parents individually (see Multimedia Appendix 2 for the interview guides). The interview sessions (approximately 1 hour) were conducted in person in participants’ homes. After the end of the interview, parents completed a brief demographic questionnaire with items on age, race, ethnicity, education level, current employment status, marital status, and housing situation. Engagement with the toy was tracked automatically by the toy throughout the deployment, by registering and logging every interaction with a timestamp.

The semistructured interview conducted at the end of the deployment included questions designed to elicit participants’ views and experiences of using the toy as well as their expectations of long-term outcomes if they were to keep it for longer. During the interviews, the photos children took and the completed activities in their discovery books were used as prompts to ask families about the child’s engagement with the toy. The interview sessions (approximately 1 hour for the parent interview and 30 min for the child interview) were conducted in person in participants’ homes when the researcher visited the families to collect the toy and accompanying materials. Families were offered £50 compensation for their time. All interviews throughout the development were audio-recorded, with permission from the parents and children; the researcher also collected simple field notes about who was present during the visits and also detailed any additional observations that seemed important but would not be captured by the audio recordings *.*

### Data Analysis

#### Analysis of Interview Data

We decided to focus the analysis in this study predominantly on the postdeployment interviews as the existing emotion regulatory practices reported by families during the predeployment interviews were similar to those described in prior work [67] (eg, strong parental emphasis on external behaviors rather than underlying emotions, expectation of self-soothing by children, and use of disengagement and distraction as 2 main ER strategies), and postdeployment interview data were rich enough to answer the research questions. Interview recordings were transcribed verbatim by the first author and an independent research assistant and then included into an inductive thematic analysis. Following Braun and Clarke’s 6-step recursive process of thematic analysis [102], the transcripts were checked against audio recordings for accuracy and then read and reread by the first author to ensure familiarization with the data. Initial codes were then generated across the dataset. As new ideas emerged, and codes were refined while working through the transcripts, previously coded transcripts were revisited to ensure that the codes still applied. Once code application was complete, resulting in 603 coded passages and 2226 code applications, different codes were sorted into potential themes by the first and fourth authors, which were then refined to generate an initial thematic map of the analysis. The refinement of the thematic map involved several iterations until authors agreed that the final themes and subthemes told a coherent story about the data. To protect anonymity, participants are referred to by using P for parents and C for children, followed by a participant number.

#### Analysis of Log Data

The prototypes logged every interaction throughout the deployment. Due to Arduino limitations, the sampling rate differed depending on the quickness of the *heartbeat* as the sensors were polled in between every 2 beats: the sampling rate was about 2 Hz in the *anxious* state and about 0.7 Hz in the *happy* state. The first author kept a detailed log about the time and date when the toys were introduced and removed from the families. The resulting log files (approximately 4.5 million lines) were then processed in R, post deployment. It is important to note here that as the data only represent activation of the toy’s sensors (on its back, ears, feet, or gyro), interpretation is limited: for instance, if the toy was moved from one place to another, or placed in a bag to be transported, a sensor could be unintentionally activated by the pressure. To partially mitigate such *accidental activations*, we have removed minutes with less than 20 separate sensor signals from the analysis.

## Results

### Demographics

The study included 11 children from 10 families as a pair of siblings received 1 toy each during the same week (female children n=3; female parents n=11; mean age of children 7.1 years [SD 1.22, range 6-10]; mean age of parents 37 years [SD 5.36, range 28-44]). For a more detailed description of participants’ demographic characteristics, refer to Multimedia Appendix 3). One additional family had a malfunctioning prototype and has been removed from the main analysis. Table 1 includes individual information for age, gender, and other deployment-related information for each of the children. We had no attrition; all participants finished all phases of the study.

**Table 1:** Overview of child demographics and the labels they associated with the prototype.

Child	Age (years)	Gender	Toy’s name (gender)
C1	6	Male	Jade/Pipsqueak (female)
C2	6	Female	Coco (male)
C3	6	Female	Winter (female)
C4	6	Male	Mr Scared (male)
C5	7	Male	Frankie (male)
C6	7	Male	Creature (female)
C7	8	Female	Rainbow (female)
C8a	7	Male	Wootie (female)
C8b	10	Male	Missy (female)
C9	7	Male	Happy (male)
C10	8	Male	Buddy (male)

### Qualitative Results

#### Engagement and Appropriation

**Figure 3:**
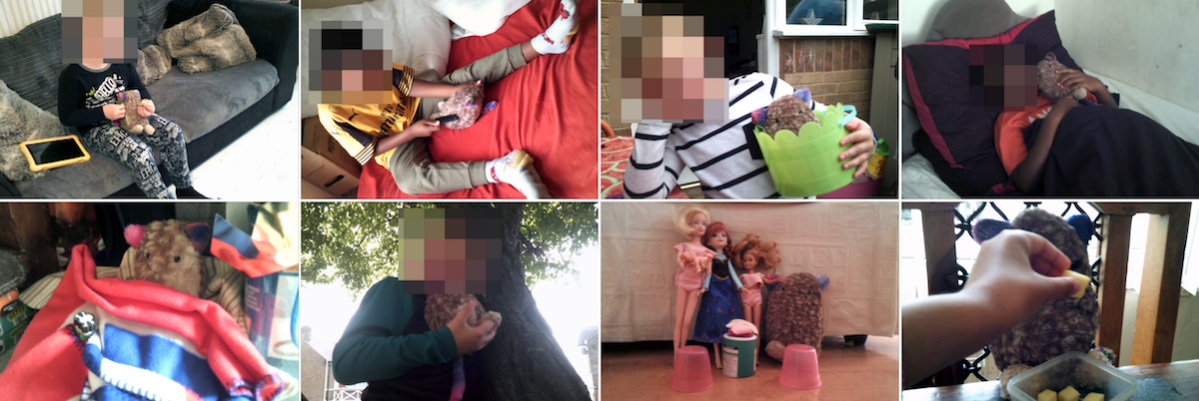
Example photos taken by children.

In describing their experiences over the week, all the children (11/11) outlined how the toy became included in their everyday routines, whether these were cuddling and stroking the toy when watching TV, playing with their other toys, or going to bed, or more active play such as role play scenarios (see Figure 3 for example photos taken by the children). For most children (10/11), their parents or themselves reported that they wanted to carry the *creature with them wherever they went* and were keen to show it to family and friends. Every child named their toy and treated it as a living being that needed to be cared for, with feelings and mental states they seemed to take into consideration. For example, most children (7/11) were very protective of the toy and looked after *its feelings*, for example, by making a bed for it to sleep in or clothes so that it would not be *cold*, making sure to soothe it when it was getting *stressed*, and being very particular about how others could interact with it in fear that they would *stress it*, break it, or take it from them. These findings are illustrated by quotes mentioned below; Multimedia Appendix 4 then provides a much more extensive set of quotes pertaining to each of the themes throughout the results section.

They were like instantly connected. Everywhere she went, she’d hug him, she spoke to her dad about Coco, to her grandmother, to her cousins. Very proud.

P2

Creature goes wherever [my child] goes...Creature comes to bed, Creature sits with us at dinner, Creature watches his tablet, Creature does just everything does. Even if we go shopping, we come to mum, creature has to come!

P6

Another indication of the children’s emotional connection to the toy was that every child was sad to part with the toy, as was reported either by children themselves or by their parents. Beyond the interview data, this was also experienced by the first author during her visits to pick up the toy, when most children would ask to keep the toy for longer or would hide it and pretend they did not know where it was. Seeing these strong impacts with the first 3 children, we decided to make repeated checks with the parents (at about a week and then 4 weeks post deployment) to make sure this was only a transient state, as well as slightly alter the narrative when deploying the toy to add that the *creature* would be returning to its family at the end of the week. We presumed that this framing would resonate with children and make it easier for them to part with the toy, thus lessening the emotional impact of the separation. Parents did not report any persisting issues during the phone checks; instead, they emphasized that children had fond memories of the toy and would still occasionally mention it:

[It was really sad when] I didn’t have it today. [...] It’s because I really loved it. And now I can’t even have it for more days.

C1

Children’s sustained engagement with the toy appears to stem from the enjoyment they gained from the in-the-moment interaction. All the families reported that interacting with the toy had a positive impact on children’s mood; a finding that is discussed in more detail in the following section. In addition, more than half of the parents (7/10) highlighted the sense of responsibility the back-story instilled in children as something that children really enjoyed and that in turn drove consistent engagement over the week-long deployment:

When I tried calming the creature down, I felt...I felt like I was actually doing something useful.

C8a

#### Impact on Emotion Regulation

Both parents and children reported that the toy was incorporated into the children’s emotion regulatory practices in a number of contexts, and all the parents recognized that the interaction with the toy had a calming effect on their children. Common observations included children naturally interacting with the toy to self-soothe after an emotion-eliciting situation, such as a conflict with their parents or siblings, or in moments they wanted to relax, such as before bedtime. Although these emotion regulatory effects were most commonly observed in situations where children were particularly upset or angry, it was also reported that having the toy had an overall calming effect, with children appearing *a lot calmer* or *more settled* over the duration of the deployment. These parental reports were complemented by the child interviews: A total of 10 out of the 11 children deliberately used the toy to calm down and reported that soothing the toy had a positive effect on their mood, making them feel *happy* or *calm*. A total of 4 children also used the toy at times they were in physical pain and described how this helped them cope with it:

Mum: What did [your sibling] do? Did you just go in the bedroom this time and he told you off? C: He told me to get out, that it’s not mine. M: Okay. But it is your bedroom. R: Oh, so that made you angry? (child nods) R: What did you do afterwards to calm down? C: I ran in, got past [sibling’s name] and started stroking the creature and hugging it. R: And how did that make you feel? C: Really happy. Overjoyed I would say.

C10

When my mum was brushing my hair...it hurts, so I usually have the creature by me so it can distract me from the pain.

C8b

Although children seemed to engage with the toy naturally during emotional moments, half of the parents (5/10) also mentioned instances where they would explicitly encourage their children to use it to soothe themselves. Only 1 parent (P1, quote below) mentioned that the toy was not on their or their child’s mind in highly emotional situations such as meltdowns, and they thought the child needed to cool down first before they could interact with the toy in a calm manner:

I saw her looking after Winter, hugging Winter, calming Winter down, using it to calm herself down. [...] Especially like when she got angry. I’m like (speaking softly) “Go and get Winter”. [...] So, yeah, sometimes I’ll direct her, sometimes she will just do it herself.

P3

I know a problem is that sometimes when they’re angry it’s not really the first thing that comes to head. Because, you know, when a child is angry, they’re angry! Do you understand? Maybe it’s just when they cool down, then that’s when they might think “you know what? Let me...” (imitates stroking movement). And then that’s when they start cooling down even more.

P1

#### Parental Views on the Causes of Observed Effects

Some parents made their own inferences as to how the toy worked to help their children calm down. Most (6/10) reported that the toy was comforting for children, with a few drawing a comparison between the toy and their children’s comfort objects, that is, items they cherished and used to comfort themselves when younger, such as blankets or soft toys. A total of 2 parents and 1 child described how the sense of responsibility children felt for the toy made them shift their attention to caring for it rather than focusing on what might have been upsetting them, thus serving as a distraction. One parent (P6) thought the toy gave her child a sense of control over the toy’s emotions that he was usually lacking in himself; the child’s account seems to support this claim as he mentioned that he liked deliberately stressing the toy so he could soothe it and himself in doing so:

Because my mind was on her, and calming her down...like she was a child to me. Because when I’m calming her down...technically my mind is completely on her...So I’m technically blocking out everything and trying to keep my child safe!

C8b

Parent: It’s something that I think...Like I said, he can control to an extent. Obviously, he can’t control when it gets upset. But it’s something that he has control over, because he doesn’t have control over those specific emotions in him. [...] So it’s the one thing that he can’t control in himself, but he can control in something else. And I think, that really worked with him...I really do.

P6

Child (independently): We can do this (cuddles the toy) and do this (presses toy’s ears) if he just keeps purring and you want him to get mad and then make him purr again. I like calming him down...because when he’s just purring it’s just...it makes me calm.

C6

Interestingly, parents’ accounts suggest that, in their view, the toy’s impact on ER was not limited to children. Half of the parents (5/10) reported that they found the interaction with the toy calming for themselves or other members of the family too, such as younger siblings or other adults.

Definitely, it can help both the mother and the child. Definitely. Which is a good thing because sometimes, some toys, people just create them just to help the child. But then, knowing there’s something that can help the adult as well, it’s even a plus! Because the same way a child needs help, the adult needs it as well. Because we get mad as much as they do! [...] It’s nice to know that there’s something that can help both!

P1

#### Parents’ Acceptance of the Intervention

Parents reported that the toy had met—or in some cases even exceeded—their initial expectations and did not have any negative feedback to relay. Parents’ accounts suggest that they held positive views of the toy and enjoyed their experience of having it at home. Notably, a parent (P4) who was initially skeptical about her child’s interest in the toy and expected that it would quickly wane described how surprised she was with her son’s strong attachment to the toy and how caring he was with it. Finally, almost every parent (9/10) reported that they would like to keep the toy at home for longer if possible and inquired if and when it would be made available to the public. Most parents (9/10) thought that the toy would continue to be a valuable resource for the children as *somewhere they could go to* to calm down when needed:

I’m impressed! I didn’t think it would be the way it has. And I didn’t expect the attachment. Really, really didn’t. Especially him being a boy and being six. [...] I personally wouldn’t change anything. I think it’s great the way it is. There’s nothing I can say “Oh, you should add this, or take away that” [...] Because it’s worked!

P4

I liked being able to refer to it, like when it was needed. And sometimes I just liked... hugging him! (chuckles) Or like seeing [my children] hug him. [...] I’ll be quite sad to let it go (chuckles). Cos you’d think they’re quite inanimate, but they’re also quite giving!

P9

### Quantitative Log Data Results

In this section, we are reporting on the interaction data automatically collected by the toy during the deployments. As outlined in the Data Analysis section, we classify any given minute as *active* only if the toy logged at least 20 different sensor interactions during that 1-min interval. This is to avoid counting accidental touches, or just moving the toy from one place to another.

**Figure 4:**
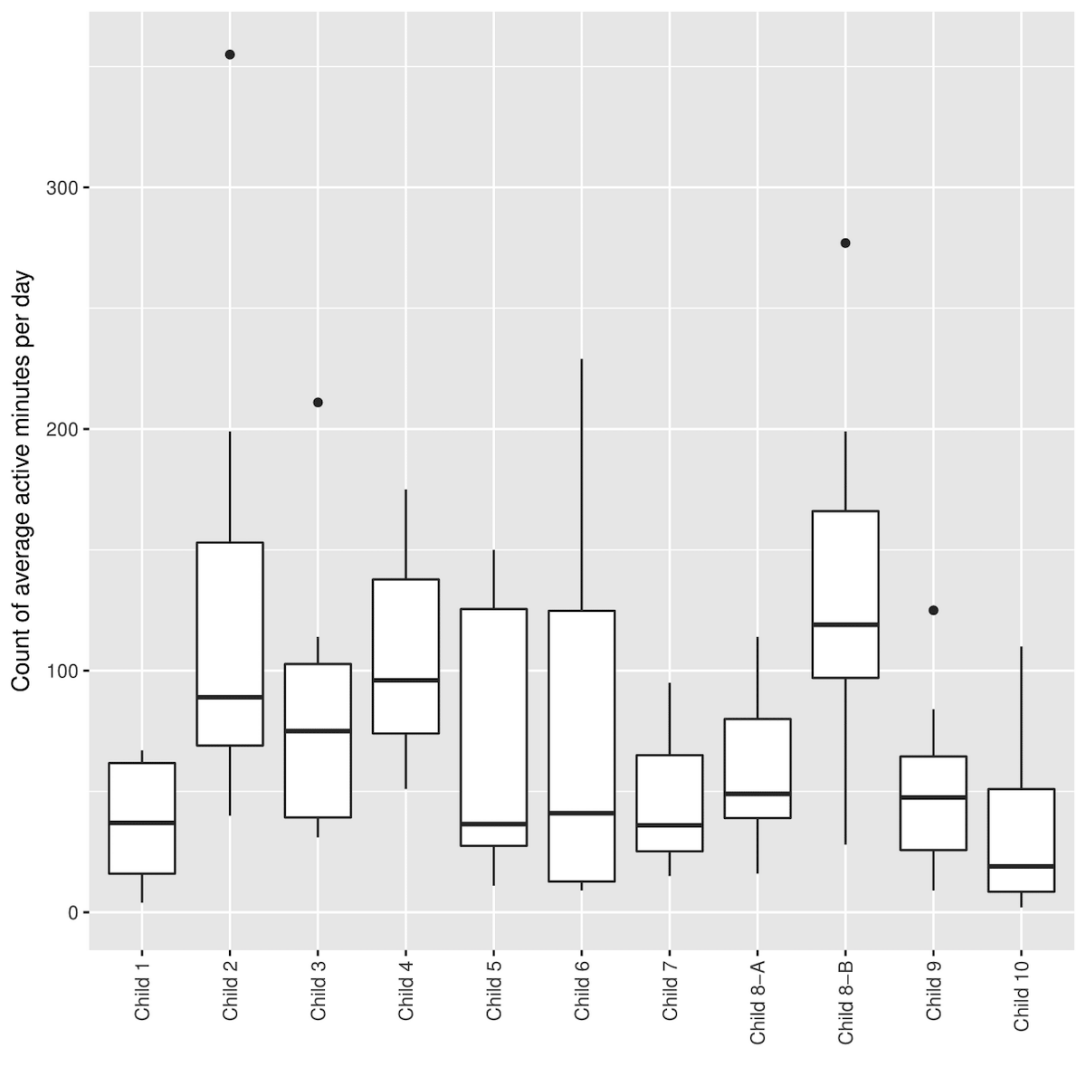
Box plots for active minutes of interaction per day for individual children.

Overall, the log data support the qualitative observations, showing sustained engagement throughout the deployment: the families used each toy, on average, for 74.9 active min per day (median 60.5; SD 64.1; see Figure 4 for box plots for individual children). We did observe that, overall, the average interaction times per day were longer for the first 3 days of the deployments compared with the last 3 days—but even then, the average active engagement was 43.8 min per day (median 30.5, SD 35.7). This might indicate that the engagement was stronger in the first few days because of novelty effects, and the children’s interest in the toy started to wane toward the end of the deployment. Another plausible explanation that would be in line with interview data is that in the first days, children and other family members interacted more with it as they were exploring the features, whereas in the last 3 days, the children already knew how the toy worked and used it as and when they needed it. Long-term deployments are needed to understand the stability of engagement beyond the first week.

**Figure 5:**
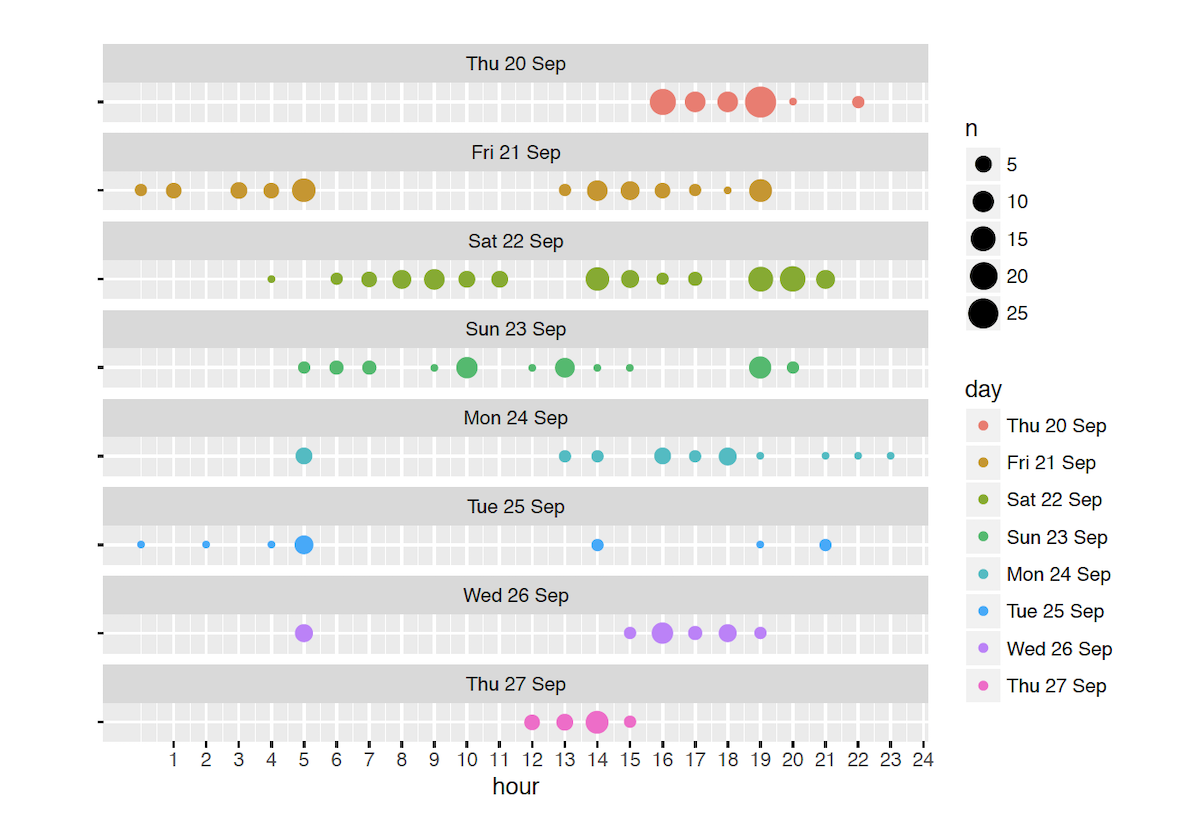
Example day-to-day summary for a child (child 7).

As expected, we observed a stronger engagement on weekends and holidays when most children would interact frequently with the toy throughout the day, whereas on school days, children interacted with it the most early in the morning (before school) and in the afternoon. To illustrate this, Figure 5 visualizes the weekly active minutes for child 7, selected as a typical example: child 7’s overall active minutes length is close to the median of the dataset and also qualitatively typical to the interaction patterns we observed for other children. In this case, comparing the data on a weekend day (Sat 22nd) and on a school day (Wed 26th) exemplifies how the active times have been influenced by school times: with the child having frequent interactions with the toy from the morning up to the evening on the weekend day, while briefly engaging with the toy in the morning before school and throughout the afternoon after their return on the school day. The log data also seem to confirm participants’ reports that children would at times interact with the toy around bedtime to relax. In some cases, interactions were also registered at nighttime, suggesting that children had the toy in bed with them; because of the inherent limitations of the log data in terms of interpretability, we cannot ascertain if these touch traces represent intentional (eg, children waking up in the middle of the night and stroking the toy) or accidental interactions.

When all interaction data are aggregated, the most frequently activated sensor was that of the back (35%), followed by the gyroscope (26%), feet (20%), and ears (20%). The large percentile of back sensor activation is consistent with the patterns of interaction reported in participants’ interviews, as hugging and stroking the toy’s back—both of which would activate the back sensor—were reported as children’s preferred soothing interactions. Although the percentile of gyroscope activation was higher than we expected, considering it consistently happened alongside the activation of the back sensor, it does not seem likely that it indicates shaking or rough handling by the children.

## Discussion

### Principal Findings

The aim of this qualitative in situ study was to investigate the engagement, acceptability, and initial subjective indicators of emotion regulatory effects for a proof-of-concept intervention model, as instantiated in a *smart toy* prototype. The novelty of the proposed approach was to deliver at-home interventions through an interactive object that becomes incorporated into child’s everyday interactions to provide in-the-moment regulatory support, without any explicit training necessary for the child or the parent.

The fundamental assumptions underpinning the logic model of such *situated and child-led* intervention was that (1) children would be naturally compelled to keep interacting with the intervention without external guidance; (2) it would become incorporated into their everyday emotion regulatory practices, even without any formal training; and finally (3) the intervention will be perceived as acceptable to parents. The qualitative findings described above suggest that all 3 conditions were satisfied: all children reported sustained engagement with the prototype, without any externally imposed conditions and have been consistently labeling such interactions as subjectively pleasing. Both parents and children further described the observed emotion regulatory effects of child-toy interaction under a variety of contexts (eg, self-soothing after an interpersonal conflict, reduction in subjective anxiety levels, relaxation support, and coping with pain). Finally, all children and 9/10 parents were keen on keeping the prototype for longer, suggesting a high acceptability and suitability with respect to social practices in the home.

The qualitative findings also provided some indicative support for the hypothesized mechanisms underpinning the first 2 levels of the logic model: level 1 as facilitating in-the-moment regulatory support (relying on attentional deployment and response modulation) and level 2 as scaffolding ongoing engagement (through the creation of an emotional attachment to the toy).

For level 1, the experiences described by both parents and children supported the in-the-moment regulatory effects: the children described the moments of holding the prototypes as *happy* and *calming*, and some have reported to deliberately seek the interaction to calm down. Interestingly, half of the parents have described similar soothing experiences themselves, suggesting that the effects might be consistent across a wider age range, as could be expected given the reliance on fundamental emotion regulatory mechanisms [68,71,73,76,77]. Although it is impossible to disentangle the assumed attentional deployment and response modulation mechanisms based on the retrospective interview data, the stories captured in the interviews provide some support for the hypothesis that physiological effects arise from a combination of tactile stimulation (eg, “I just put it to my chest and it worked” type of quotes common across the dataset) and more conscious focus on *changing the creature’s emotions*.

Similarly, the hypothesized level 2 mechanisms have received indicative support in the interview dataset. All children referred to the prototype as if it were alive, attributing a range of human-like mental states to the toy, together with an associated range of caring behaviors (eg, making a bed or custom-made clothes to help it *feel warm*, making sure it is not *stressed*, and controlling how others interact so as to not *hurt it*). Combined with the sadness associated with the end of deployment, these observations suggest that the prototype was successful in generating an emotional attachment, which appeared to facilitate the continued engagement. These relationship-building effects appear analogous to those observed with other animal-like robots in other contexts: see Turkle et al [103] for a critical analysis of the mechanisms behind such computational devices presenting themselves as *relational artifacts*.

The study data do not provide indications of any effects on longitudinal shifts in emotion regulatory practices (level 3) because of the short-term deployment and lack of baseline and follow-up measurements. Further efficacy research, including in situ studies (such as randomized wait-listed designs in schools), is needed to understand the effects of the existing prototype on child ER practices and mindsets. Interesting research directions also include questions around the impact of associated materials (such as the discovery book) on the intervention effectiveness.

### Similarities and Differences to Existing Interventions

To the best of our knowledge, the proposed intervention model is unique in prevention science as it suggests an intervention delivery method that becomes fully embedded in children’s everyday lives, does not require any explicit training, and is relying on in-the-moment experiential support rather than information delivery. It draws inspiration from the large body of research on animal-assisted interventions (see Crossman [104] for a review), which has suggested promising outcomes in a number of populations. These include increased social interaction among children with autism spectrum disorder [105], increased social behaviors and reduced agitation and aggression among persons with dementia [106], reduction in symptoms among patients with depression [107], and increased emotional well-being such as reduced anxiety and fear [108]. A related area of work is focused on *social assistive robots* [84,105-108], which are designed to act as pet surrogates, such as the robotic seal Paro [109]. A majority of such socially assistive robotics (SAR) interventions has so far, however, focused on occasional use by older adults, particularly those suffering from dementia [109-114].

The design of SAR with typically developing children has been limited to educational interventions outside of mental health domain [115-117]. Despite the reported promising outcomes of SAR interventions in other contexts, no studies to date explored the use of SARs as part of prevention interventions (for ER or other protective factors) with typically developing children, and only 1 recent study [80] has explored the effects of interacting with Paro robot on children’s mood, anxiety, and arousal after exposure to a lab-based, stress-inducing task: interaction with the robot resulted in greater increases in positive mood than any of the control conditions but did not have a significant effect on negative mood, anxiety, or arousal.

### Broader Implications: Potential for Situated and Child-Led Interventions

More broadly, this proof-of-concept prototype can be seen as illustrative of a conceptual shift in how early prevention interventions might be created and delivered with technology: the notion of *situated interventions* and *child-led rather than parent-driven* approach.

The goal of a *situated intervention* refers to designing programs that will allow the families to draw on—and learn from—specific lived experiences as part of the intervention. This goes beyond purely *just-in-time* intervention delivery such as reminders or activity suggestions [37,118]: the purpose is to flip the existing intervention model that is based on information delivery and didactic learning (eg, at an in-person workshop or classroom lesson) *to be applied later* toward a model where the intervention directly supports both children and parents to learn from the daily emotional challenges they encounter. As with the example prototype discussed here, successful situated interventions would aim to embed intervention delivery as an implicit part of everyday situations—such as those of stress, anxiety, or sadness in the case of the toy presented in this study. The goal is then to utilize these everyday moments as an opportunity for ongoing, iterative training, rather than having to rely on vignettes, role-plays, or the recollection of past experience as is common now [18,22,34]. Psychologically, the notion of *situated interventions* thus corresponds to the need for in-the-moment scaffolding of experiential learning that underpins all socioemotional competencies [22,51,119-121] but has been pragmatically impossible to date.

The second key shift toward *child-led* interventions argues for the potential of repositioning the child as the immediate recipient of some or all aspects of the technology-enabled intervention. In the current prevention science models, the child is either seen as a *captive audience* within the in-school programs or as a secondary actor who is impacted by parental training. The reasons for this are understandable: the existing interventions could not rely on young children to drive the intervention as it is, for example, unlikely that a child aged 6 years would be able to teach their parents new parenting strategies as a workshop coach might, or directly engage (or want to engage) with a written text on a leaflet sent home. The ongoing, in-the-moment scaffolding facilitated by situated, technology-enabled interventions could address both of these issues and reposition the child as the main actor of the intervention, both in terms of who is driving the intervention transfer to home as well as who is to be engaged with the intervention once it is there.

### Strengths and Limitations

One strength of the study was the emphasis on in situ unstructured deployments, which provided ecologically valid data about possible appropriation in families. Most parents were from underprivileged neighborhoods, and many were in difficult personal situations; we have avoided tapping into the proverbial *worried well* and instead worked with a population who could be expected to strongly benefit from ER interventions [122-124]. The detailed interviews then provided a holistic understanding of how the prototypes have been used and the impact they might have on the family life. Another strength was including the interview data from both parents and children (in addition to photographs collected by participants during the week), triangulating the evidence across all stakeholders.

The data have been promising in terms of observed engagement and acceptability, which were high across all 10 families recruited into the study. This consistency—together with analogous positive effects from earlier deployment [67]—is particularly promising in view of the commonly high attrition rates and nonengagement for technology-enabled mental health interventions [10,38-41]. However, there may have been some self-selection recruitment effects: the families have explicitly opted into the study and, thus, might be more likely to respond positively than the *general* population. Further studies should investigate the engagement rates when deployed, for example, as part of school-based approaches and with reduced researchers’ engagement (eg, questionnaire rather than interview methods).

An expected limitation of a pilot qualitative study is the lack of definitive data on psychological effects. Although participants’ reports suggest that they experienced subjectively significant changes to their everyday emotion regulatory practices, more rigorous studies are necessary to understand the strength of psychological effects and whether these would scale up. In particular, it is not yet clear if these would lead to long-term changes, and whether the magnitude would lead to a clinically significant change in emotion-coping mechanisms and strategies [45]. As such, the lack of data on the presumed level 3 effects is the most important gap. It will require not only rigorous efficacy study designs to estimate the current effects but also likely further iterative codesign development (with parents, children, and prevention science experts) to strengthen the intervention impact. The qualitative pilot data from this and previous publication [67] provide a good starting point for such future work.

### Conclusions

This is the first known study investigation of the use of object-enabled intervention delivery to support ER in situ. To understand the feasibility of such novel intervention mechanism, this qualitative study examined its appropriation and engagement by 11 children from low-socioeconomic status families over the period of 1 week. Triangulating both parental and child interviews, the data provide a holistic picture of how the prototype was incorporated into the family life. The strong engagement and qualitative indications of effects are promising—children were able to use the prototype without any training and incorporated it into their ER practices during daily challenges. Future work is needed to extend these indicative data with larger studies examining the psychological efficacy of the proposed intervention. More broadly, our findings suggest the potential of a technology-enabled shift in how prevention interventions are designed and delivered: empowering children and parents through *child-led, situated interventions*, where participants learn through actionable support directly within family life, as opposed to didactic in-person workshops and a subsequent skills application.

## Appendix

Multimedia Appendix 1 Design of the smart toy prototype and detailed logic model.

Multimedia Appendix 2 Interview guides.

Multimedia Appendix 3 Demographic profile of participating parents.

Multimedia Appendix 4 Summary of themes and illustrative quotes.

## Abbreviations

ER: emotion regulation
SAR: socially assistive robotics
UCL: University College London
