# Supplementary material for: A Smart Toy Intervention to Promote Emotion Regulation in Middle Childhood: Feasibility Study
Source: JMIR Ment Health. 2019 Aug 5;6(8):e14029. doi: 10.2196/14029 (PMC6699114; doi:10.2196/14029)
Supplement: Multimedia Appendix 1 [file mental_v6i8e14029_app1.docx]

Appendix 1: Design of the smart toy prototype and detailed logic model.

**Physical design**

The prototype takes the form of an animal-like furry creature with eyes, a spotty brown body, multi-coloured ears and tail, and light brown feet. We posited that a small, animal-like form would be appealing to children and inviting to petting-style touch interactions. We made the explicit design choice not to have it resemble any existing animal, so as to avoid any aversions children might have to particular animals, but also to allow children to use their imagination to personalise the narrative around the creature. The remainder of this section will detail the prototype's hardware design.

**Body**: The main boards are housed in a plastic box inside the toy. We chose to use an Adafruit Feather M0 board with an on-board SD card and included a Real-Time Clock (RTC) to allow for extended data capture with accurate time stamps. We included a gyroscope and used a moving average filter to detect when the toy was picked up or if it was being shaken too much: slow movements would go unnoticed by the creature, but quick changes would send it into an anxious state (see next section for more details).

To detect any petting/stroking motion on the toy’s back, we added a capacitive touch peripheral board. To make it look and feel like a natural part of the creature's fur, we fringed a few strips of the conductive fabric and inserted it into the centre back seam.

**Ears:** The prototype’s ears contain mechanical click buttons embedded in soft sheet foam. The idea was that this would allow for an interaction which would feel like manipulating an animal’s cartilaginous ears.

**Feet:** The feet contain a circular Force Sensing Resistor (FSR) positioned between poly-bead fill to create a smooth rolling texture. The idea here was to offer this as a ‘foot massage’ style engagement with the creature.

**Tail:** The tail is supported by firm stuffing and a stiff wire to retain its shape.

**Interaction design**

In terms of the interaction design, the `creature’s’ internal state is rendered through the vibration patterns that mimic a heartbeat, with faster rates corresponding to higher stress levels. The creature’s internal state directly corresponds to a simple counter, which goes up or down depending on the haptic interactions registered by the various sensors. We categorised positive and negative interactions among the sensors, which either increased or decreased the counter and, thus, the heart rate. Stroking the creature’s back or massaging its feet were classified as positive/soothing, whereas giving it a hard shake or clicking its ears were classified as negative/distressing. For example, if the child pressed one of the feet or stroked the toy’s back, then the counter would get +1, but if they clicked one of the toy’s ears, the counter would get -5. The counter values were capped at -15 and +45.

The counter has 5 different `action modes’, each corresponding to a different internal state and vibration pattern:

- **very anxious** state (~130bpm) counter < - 10
- **less anxious** state (~120bpm) counter < +15
- **neutral** state (~108bpm) counter < +25
- **calm** state (~100bmp) counter < +30
- **deeply satisfied** state (purring sound) counter < +40

The creature wakes up with a fast `heartbeat’ and the child has about 1 minute to soothe it before it falls back asleep in this stressed state. If the child successfully soothes the creature with 15 touch interactions within that minute, this extends the internal counter by an additional 30 seconds, and the creature’s heartbeat slows down. If the child provides an additional 10 interactions, the creature shifts to a purring vibration and stays in that mode for one more minute before falling asleep.

**Logic model**

The proposed logic model underlying the intervention is assumed to operate on three levels building on each other: (i) the first level pertains to directly providing in-the-moment soothing support to children in naturally occurring emotional moments when they would attempt to calm down; (ii) the second is concerned with mechanisms that facilitate children’s longer-term engagement with the intervention, providing meaning and motivation for ongoing use; while (iii) the third is assumed to emerge from repeated experience of soothing interactions over time, leading to a shift in children’s emotion regulation practices and implicit beliefs about emotion. The present study aimed to provide pilot indicative data pertaining to levels #1 and #2.

**Level 1 – In-the-moment support** The prototype’s physical and interaction design was aimed to tap into a number of known regulatory factors, grounded theoretically in Gross’ extended process model of emotion regulation [65]. This model categorises emotion regulation processes depending on the point in the emotion-generative process at which they operate. We designed the prototype interaction with the aim to impact two separate stages: the attentional deployment stage, by shifting children’s attention from the emotion-eliciting situation towards interacting with the toy; and the response modulation stage, by facilitating down-regulation through pleasant tactile interaction. Due to the temporal dynamic and circular nature of the emotion regulation process over time, the two stages are expected to be functionally linked and support each other in nudging the child towards reduction of negative emotions at each instance of the regulatory circle. Such sequenced strategies have been previously shown to be effective (cf., [94]) and are commonly observed in practice [68,87]. The specific research grounding and design choices supporting the individual mechanisms were as follows:

**Attention deployment:** Research on emotion regulation choice has shown that in high-intensity situations, individuals prefer diverting their attention from emotional stimuli, possibly because disengaging attention early on provides short-term relief with minimal effort by successfully blocking emotional information at an early processing stage before it gathers force [71-73]. We hypothesised that the interaction with the prototype could support such attentional re-deployment: by focusing on the ‘toy’ and its interactive responses to their manipulation, children would distract themselves from the emotional situation they were encountering. Shifting and maintaining attention away from the negative emotional triggers can possibly disrupt cognitive elaboration on the negative events (i.e., rumination) and allow the negative emotions to subside [74]. The framing of ‘taking care’ of the pet was assumed to provide further motivation and meaning for the child-toy interaction (see [75]), thus acting as a secondary, cognitive mechanism facilitating attentional deployment.

- **Response modulation:** Research across a range of domains suggests that tactile stimulation may be one of the adaptive response modulation strategies in buffering stress responses: this mechanism is assumed to underpin the emotion regulatory effects of human-animal interaction (cf. [76] for a review), play a role in effects of ‘social touch’ [77-79], and the soothing effects of animal-like robots [80,81]. Based on this literature, we hypothesised that the interaction with the prototype could also tap into the response modulation stage by affecting the child’s physiological emotional response.

**Level 2 – Ongoing engagement** The second level then directly builds on the positive subjective experience of in-the-moment soothing to facilitate long-term engagement with the toy through the associated narrative. The framing of the toy as an ‘anxious creature in need of assistance’ is the hypothesised key driver: we assume that this framing will not only support convey the benefits resulting from extrinsic emotion regulation [77,82,83], but also facilitate the creation of a sense of relationship and responsibility for the ‘well-being’ of the creature, similar to the long-term engagement seen with child-oriented robots (e.g., [75]) or products such as Tamagotchi [84-86]. If the design and associated narrative are successful in creating an emotional attachment to the toy, we expect that children will treat it as a being with thoughts and feelings [95], which then provides another layer of meaning to their interactions, facilitates the incorporation of the child-prototype interactions throughout everyday situations, and further motivates sustained engagement.

**Level 3 – Shift in emotion regulation practices and implicit beliefs about emotion** Finally, we envision that repeated interactions with the toy will result in the establishment of more adaptive emotion regulation patterns and shift children’s implicit beliefs about the controllability of emotion [87,88]. Specifically, we hypothesise that the repeated experience of in-the-moment soothing will influence children’s implicit beliefs about emotion over time, leading them to evaluate emotions as more controllable (as they are consistently regulating these with the help of the toy), and therefore themselves as more equipped to cope. Beliefs about the controllability of emotion are considered a prime target for intervention as they have implications for emotion regulation [89-93]. They are of critical importance both for acute (e.g., emotional experiences), and more chronic, cumulative outcomes (e.g., well-being): numerous studies have found that the belief that emotions are uncontrollable is correlated with worse psychological health [96-98], and predictive of future depressive symptoms [89,99]. Moreover, enabling children to assume the role of caretaker and ‘regulator’ for someone else (through the attribution of emotions to the toy) might provide them with opportunities to practise and enhance their emotion regulation skills, which they can then apply to future situations where they themselves need to calm down. This is consistent with prior research, suggesting that helping others regulate their emotional reactions can improve one’s own regulatory skills and emotional well-being [77,83]. As these effects are expected to arise only through on-going long-term interactions and thus rely strongly on appropriation in-situ, we did not expect to see any indicative data for these proposed mechanisms within this pilot study.
