# Supplementary material for: A Smart Toy Intervention to Promote Emotion Regulation in Middle Childhood: Feasibility Study
Source: JMIR Ment Health. 2019 Aug 5;6(8):e14029. doi: 10.2196/14029 (PMC6699114; doi:10.2196/14029)
Supplement: Multimedia Appendix 2 [file mental_v6i8e14029_app2.docx]

Appendix 2: Interview guides

**Pre-deployment parent interview guide**

1. How many children do you have and what are their ages and gender?
2. What are some events that cause your child the most distress, anxiety, frustration? Can you give me an example?
3. How can you tell when your child is **sad**, what do they do?
   1. Can you tell any **subtle signs**?
   2. What **strategies** do you use with your child to help them calm down when they are sad? What do you do?
   3. How does your child respond to that?
   4. How do you think this helps them calm down? What does it do for them?
4. How can you tell when your child is **angry** with you, what do they do?
   1. Can you tell any **subtle signs**?
   2. What **strategies** do you use with your child to help them calm down when they are **angry with you**? What do you do?
   3. How does your child respond to that?
   4. How do you think this helps them calm down? What does it do for them?
5. Is the approach you use to help you child calm down different/similar when they are **angry with someone else**? If so, how? What do you do?
   1. How does your child respond to that?
   2. How do you think this helps them calm down? What does it do for them?
6. Do you talk with them about the situation that upset them? If so, when?
7. Can you think back to the **last time your child needed to calm down**?
   1. What happened? What did they do? What did you do?
   2. What would you have liked your child to do in that situation?
      1. What would need to change?
      2. Are you already working on this?
      3. What are you doing/hoping to do?
      4. Does anyone else support this?
8. Does your child have any **toys or physical objects** that help them calm down?
   1. If so, what is it?
   2. How and when does your child use it?
   3. Does it work?
   4. How do you think it helps them calm down, what do you think it does for them?
9. Have you ever **bought** something for your child to help them calm down?
   1. If so, what was it?
   2. How and when does your child use it?
   3. Does it work?
10. How are the strategies you are using at the moment **to calm your children down** working out for you?
    1. Is there anything you find challenging? What would that be?
11. What strategies do you use **when you need to calm yourself down**?
12. How do you **recognise** that you need to calm down?
13. Do you have any **physical objects** that help you calm down?
    1. If so, what is it?
    2. How and when do you use it?
    3. Does it work?
14. Can you think back to one time **when you yourself needed to calm down**? Whether that was because of your child, or perhaps at work.
    1. What happened? What did they do? What did you do?
    2. What would you have liked to do if you could do it again?
    3. Is this similar/different to other situations?
15. How are the strategies you are using **to calm yourself down** working out for you?
    1. Anything you find challenging? If so, what would that be?
16. How important do you see **being good at calming down** is..
    1. for your child?
    2. for yourself?
17. What are you expecting/hoping to get out of having the creature at home this week?
18. How do you think your child will respond to having the creature at home?
19. Is there anything you are uncomfortable with or worried about?
20. Would you like to add anything else?

**Post-deployment parent interview guide**

1. What were your thoughts when you first saw the creature?
2. How would you describe this creature to another parent?
3. What happened when your child had the creature with them? What did your child do with it? (when; how long; where; anything they did repeatedly?)
4. Did you get to do anything with it yourself at some point?
   1. If so, what did you do?
5. Did you get to do anything together with the child around the creature?
   1. If so, what did you do?
   2. How did it feel/what did you think?
6. How do you think your child felt about having the creature?
   1. Could you give me an example of an interaction you observed and how you think that made your child feel?
7. What did your child tell you about the creature?
   1. Did they discuss the creature’s feelings with you?
8. When your child had the creature with them, was there a situation that made them upset for some reason?
   1. If so, what was it and how did they deal with it? (Prompts: What did they do afterwards; where; did they use the creature)
9. Were there any days that were particularly interesting, whether in a good or a bad way?
10. Did you notice your child behaving differently in any way this week? Whether good or bad?
11. What did you like the most about having the creature at home?
12. What did you like the least?
13. How would you feel about having the creature at home for longer?
    1. What do you think would happen?
14. Do you think having the creature at home for longer could help your child?
    1. If so, why?
15. Do you think having the creature at home for longer could be problematic for your child?
    1. If so, why?
16. Are there any ways you imagine that having the creature at home could help you out as a parent?
    1. If so, how?
17. If you were to have the creature here again, what do you think we should we change?

**Post-deployment child interview guide**

1. Did you name the creature? If so, how is he/she called?
2. Where did the creature live in your home?
3. Should we have a look at your discovery book and the photos you took now? (*use booklet and photos as prompts, asking where the photos were taken and what was happening)*
4. Did anyone else do anything with the creature?
   1. (If so) Who and what did they do?
   2. Did your parents do anything with the creature? (If so) What did they do?
5. Was there a situation that made you upset or worried while the little one was with you?
   1. If so, what did you do to calm down/relax?
6. What was your favourite thing about having the creature at home?
7. What was your least favourite thing about having the creature at home?
8. That was brilliant, thank you so much! I thought we could play a little game now, what do you think? In this hat there are pieces of paper with beginnings of sentences; you pick up a piece of paper from the hat, read what it says out loud and then finish the sentence. Shall we begin?
   - “When I first saw the creature, I noticed that …”
   - “When I showed it to my parents, they ...”
   - “What really worked to calm the creature down was …”
   - “When I tried calming the creature down, I felt …”
   - “My favourite thing to do with the creature was … because ...”
   - “The creature and I had most fun when…”
   - “I felt best when the creature and I … because …”
   - “If I were able to take it home for even longer, I would …”
   - “It was really sad when …”
