# Supplementary material for: A Smart Toy Intervention to Promote Emotion Regulation in Middle Childhood: Feasibility Study
Source: JMIR Ment Health. 2019 Aug 5;6(8):e14029. doi: 10.2196/14029 (PMC6699114; doi:10.2196/14029)
Supplement: Multimedia Appendix 3 [file mental_v6i8e14029_app3.docx]

Appendix 3: Demographic profile of participating parents

| **Variable** | **n** | **%** |
| --- | --- | --- |
| Ethnicity |  |  |
| White | 5 | 55.6 |
| Asian | 1 | 11.1 |
| Mixed | 1 | 11.1 |
| Black/African/Caribbean | 1 | 11.1 |
| Other | 1 | 11.1 |
| Education level |  |  |
| GSCEs (or equivalent) | 4 | 44.4 |
| A-levels (or equivalent) | 2 | 22.2 |
| Bachelor’s degree | 2 | 22.2 |
| Postgraduate degree | 1 | 11.1 |
| Employment status |  |  |
| Employed part-time | 2 | 22.2 |
| Self-employed | 2 | 22.2 |
| Not employed | 5 | 55.6 |
| Marital status |  |  |
| Single, never married | 3 | 33.3 |
| Married/in civil partnership | 2 | 22.2 |
| Cohabiting | 4 | 44.4 |
| Housing situation |  |  |
| Own home | 3 | 33.3 |
| Private rent | 2 | 22.2 |
| Council/Housing association | 4 | 44.4 |
