# Supplementary material for: A Smart Toy Intervention to Promote Emotion Regulation in Middle Childhood: Feasibility Study
Source: JMIR Ment Health. 2019 Aug 5;6(8):e14029. doi: 10.2196/14029 (PMC6699114; doi:10.2196/14029)
Supplement: Multimedia Appendix 4 [file mental_v6i8e14029_app4.docx]

Appendix 4: Summary of themes and illustrative quotes

| **Theme** | **Parent quotes** | **Children quotes** |
| --- | --- | --- |
| **Engagement and appropriation** | *“They were like instantly connected. Everywhere she went, she’d hug him, she spoke to her dad about Coco, to her grandmother, to her cousins. Very proud.”* [P2] | *“My favourite thing to do with the creature was [to] watch TV, read. Sleep. Play with it. Draw. Make new friends!”* [C7] |
|  | *“Creature goes wherever [my child] goes... Creature comes to bed, Creature sits with us at dinner, Creature watches his tablet, Creature does just everything does. Even if we go shopping, we come to mum, creature has to come!”* [P6] | *“She slept with me! [...] And she’s always with me.”* [C6] |
|  | *“When he comes home, he would look for Frankie. After he takes off his jacket and stuff, he comes in and he looks for Frankie and he sits down and plays with him. And then after dinner time again, <child’s name> comes and plays with him. Bedtime he said he wants to put him in bed with him. So sometimes I leave him.”* [P5] | *“He was a little bit stressed, so I moved him right here.. And I, I.. wanted to put a blanket in there for his bed. And I put a nest in there for like a little... toy.”* [C4] |
|  | *“You get very directed on how to use the creature. “Do this to the creature, don’t do that to the creature”. The second it vibrates he takes it away from you. (laughs) [...] I’m allowed to rub Creature, I’m allowed to pat Creature. But I’m not allowed to do anything else.”* [P6] | *“So I made him a little bed because he was starting to get really tired. [...] It was made out of my tie, and my V-neck jumper.. I didn’t have anything else with me.”* [C6] |
|  | *“I really liked seeing [my child] sort of follow through and want to finish the tasks. […] And I’m not sure if that was because it was something he was building a relationship with or just because he felt that responsibility and he was enjoying it.. I don’t know! I think he just really enjoyed the whole process and you could just- I could see that anyway!”* [P9] | *“Well, for the first two nights she slept in a pillow down there (pointing to a spot on the living room floor, next to an armchair). I had a blanket for her, but then I thought.. just to keep her a bit more warm, she could stay at my bed.”* [C8b] |
|  | *“It gave him something to concentrate on, really.. To look after. Even though we’ve had other things for him to look after, that.. he seems to have wanted to and enjoyed it.”* [P10] | *“[It was really sad when] I didn’t have it today. [...] It’s because I really loved it. And now I can’t even have it for more days*.*”* [C1] |
|  | | *“It was really sad when we give [sic] it back. [...] I liked it.”* [C5] |
|  |  | *“I was feeling a bit sad because she was gonna go, and I was feeling like I loved her.”* [C8a] |
|  |  | *“‘When I tried calming the creature down, I felt’... I felt like I was actually doing something useful.”* [C8a] |
|  |  | *“I was feeling like laughing, and I was happy, because I made.. Because I was taking care of her.”* [C7] |

| **Theme** | **Parent quotes** | **Children quotes** |
| --- | --- | --- |
| **Parental views on the causes of observed effects** | *“I think it was comforting for her... As I said, it was a lot like having her blankie when she’s.. It’s like a comfort... Yeah... When she came home from school, she’d grab hold of it and... [...] It sort of soothes her when she’s feeling stressed or... It just makes her feel safer I think.”* [P7]  *“Parent: It’s something that I think... Like I said, he can control to an extent. Obviously, he can’t control when it gets upset. But it’s something that he has control over, because he doesn’t have control over those specific emotions in him. [...] So it’s the one thing that he can’t control in himself, but he can control in something else. And I think, that really worked with him... I really do.  Child (independently): We can do this (cuddles the toy) and do this (presses toy’s ears) if he just keeps purring and you want him to get mad and then make him purr again. I like calming him down.. because when he’s just purring it’s just... it makes me calm.” [*P6 & C6] | *“Because my mind was on her, and calming her down.. like she was a child to me. Because when I’m calming her down.. technically my mind is completely on her.. So I’m technically blocking out everything and trying to keep my child safe!”* [C8b] |
|  | *“And this morning, as I said, it was really... it helped me. I thought, “Oh! If I hadn’t tried it, I could never believe that”. I thought that it would be a human being that could help me. I wasn’t talking to anyone as I always do. And I wasn’t praying or anything.. I just put this thing to my chest and it just worked. And I’m just telling you the truth. It did! Yeah, it did! (laughs)”* [P5] |  |
|  | *“I mean I actually enjoyed the little cuddles I had with the creatures! (chuckles) It was very calming, very.. It was a nice toy.”* [P8] |  |
|  | *“Definitely, it can help both the mother and the child. Definitely. Which is a good thing because sometimes, some toys, people just create them just to help the child. But then, knowing there’s something that can help the adult as well, it’s even a plus! Because the same way a child needs help, the adult needs it as well. Because we get mad as much as they do! [...] It’s nice to know that there’s something that can help both!”* [P1] |  |

| **Theme** | **Parent quotes** | **Children quotes** |
| --- | --- | --- |
| **Impact on emotion regulation** | *“She got upset, and then she took Coco and she just calmed down and she laid down. If she was sent to her room, she’d go up to her bed and just lay down with Coco.”* [P2] | *“C: When I tried calming the creature down, I felt.. happy!  R: Why did you feel happy?  C: Because I like petting him, hugging him!”* [C2] |
|  | *“I mean like with this toy she’s able to calm down a lot faster than like I said before, just going into her room and... tantrum and whatnot.”* [P3] | *“C: I was really sad when [my sibling] punched me in the bladder!  R: Oh, did he? Naughty [sibling]! And what did you afterwards? How did you calm down?  C: I told mummy. And gave Happy a hug!”* [C9] |
|  | *“It takes off a lot of the pressure. Having that go-to, where you know there’s something that for sure that will calm him. Because... The thing is, it’s a very hit-and-miss when it comes to calming him. With all other emotions you can kind of... settle it a bit. Like if he’s angry, you can reduce the anger by taking him out of the situation, or if you have to, by pinning him down. If he’s sad you can cheer him up... But with anxiety, it is the one that is too much for him. And no matter how anxious he gets you can’t always keep him calm, and it comes out in his behaviour. So for him, having that has kept his anxiety quite low. And even when things that happened that normally would have set him off, haven’t! Which is unbelievable to be honest. Honestly, I’d never thought I’d see something that would actually keep him quite so calm. [...] My house has been a lot calmer! (chuckles) It’s been so much more peaceful for the past week. There haven’t really been any attitude... Like... Just his whole entire behaviour has been awesome.”* [P6] | *“P: What did [your sibling] do? Did you just go in the bedroom this time and he told you off?  C: He told me to get out, that it’s not mine.  P: Okay. But it is your bedroom.  R: Oh, so that made you angry?  (child nods)  R: What did you do afterwards to calm down?  C: I ran in, got past [sibling’s name] and started stroking the creature and hugging it.  R: And how did that make you feel?  C: Really happy.. Overjoyed I would say.”* [C10] |
|  | *“Actually, he’s been good this week. Because he had a home visitor, as well, from school, and he’s.. He calmed down a lot! You know, even today at school when he went to meet his teacher, he seemed a lot more settled. So yeah, he has calmed down a lot for the past week. And I’m hoping that Frankie has something to do with it ! (laughs)[...] I think it does. Because he has him every single day. Apart from that evening when we couldn’t find him; we found him and then the following morning he straight away picked him up.”* [P5] | *“R: And how did that made you feel, when you had Creature and you were playing on your tablet?  C: I felt really happy... And then this stopped hurting, this stopped hurting, and this stopped hurting (pointing to his bruises and scratches from a fall).”* [C6] |
|  | *“I saw her looking after Winter, hugging Winter, calming Winter down, using it to calm herself down. [...] Especially like when she got angry. I’m like (speaking softly) “Go and get Winter”. [...] So, yeah, sometimes I’ll direct her, sometimes she will just do it herself.”* [P3] | *“When my mum was brushing my hair.. it hurts, so I usually have the creature by me so it can distract me from the pain.”* [C8b] |
|  | *“I know a problem is that sometimes when they’re angry it’s not really the first thing that comes to head. Because, you know, when a child is angry, they’re angry! Do you understand? Maybe it’s just when they cool down, then that’s when they might think “you know what? Let me..” (imitates stroking movement). And then that’s when they start cooling down even more.”* [P1] |  |

| **Theme** | **Parent quotes** |
| --- | --- |
| **Parents’ acceptance of the intervention** | *“I’m impressed! I didn’t think it would be the way it has. And I didn’t expect the attachment. Really, really didn’t. Especially him being a boy and being six. [...] I personally wouldn’t change anything. I think it’s great the way it is. There’s nothing I can* *say ‘Oh, you should add this, or take away that’.. [...] Because it’s worked!”* [P4] |
|  | *“[What I enjoyed the most was] just watching her care for someone. Because sometimes, I do, like, “Look after your sister, come on..” trying to develop that in her. Because I feel that it hasn’t, you know, that kind of caring side? But I saw she was able to do that with Coco. So I was like “Okay, she’s very caring, and compassionate”.”* [P2] |
|  | *“I liked being able to refer to it, like when it was needed. And sometimes I just liked.. hugging him! (chuckles) Or like seeing [my children] hug him. [...] I’ll be quite sad to let it go (chuckles). Cos you’d think they’re quite inanimate, but they’re also quite giving!”* [P9] |
|  | *“If they were on sale, I would happily buy one for him, really. I honestly do think it’s such an amazing idea. [...] He just seemed to be coping better with him.”* [P6] |
|  | *“Knowing that there’s a toy that is calming and soothing for them, to remind and say if they are getting upset or worked up or something, would be quite useful. Like when we said “Look, let’s turn around and go and get your creatures”. So a particular toy that’s associated with calming them down and they can care for and look after.. and that would purr and the feeling of that warmth and the realness of them close to them.. I think if there are difficult times it would be useful.”* [P8] |
